# High Pregnane X Receptor (PXR) Expression Is Correlated with Poor Prognosis in Invasive Breast Carcinoma

**DOI:** 10.3390/diagnostics11111946

**Published:** 2021-10-20

**Authors:** Stamatios Theocharis, Constantinos Giaginis, Stefania Gourzi, Paraskevi Alexandrou, Gerasimos Tsourouflis, Panagiotis Sarantis, Eugene Danas, Artemis Michail, Nikolaos Tsoukalas, Alexandros Pergaris, Panagiotis K. Politis, Lydia Nakopoulou

**Affiliations:** 1 First Department of Pathology, Medical School, University of Athens, 11527 Athens, Greece; stefaniagourtzi@yahoo.com (S.G.); parialexandro@yahoo.gr (P.A.); gtsourouflis@med.uoa.gr (G.T.); psarantis@med.uoa.gr (P.S.); danaseugene@gmail.com (E.D.); tsoukn@yahoo.gr (N.T.); alexperg@yahoo.com (A.P.); lnako@med.uoa.gr (L.N.); 2 Department of Food Science and Nutrition, University of the Aegean, 81400 Lemnos, Greece; cgiaginis@aegean.gr; 3 Section of Molecular Biology, Biomedical Research Foundation, Academy of Athens, 11527 Athens, Greece; artemisbio@gmail.com (A.M.); ppolitis@bioacademy.gr (P.K.P.)

**Keywords:** PXR, breast cancer, immunohistochemistry, clinicopathological parameters, patients’ prognosis, cell lines

## Abstract

Pregnane X Receptor (PXR) is involved in human cancer, either by directly affecting carcinogenesis or by inducing drug-drug interactions and chemotherapy resistance. The clinical significance of PXR expression in invasive breast carcinoma was evaluated in the present study. PXR protein expression was assessed immunohistochemically on formalin fixed paraffin-embedded breast invasive carcinoma tissue sections, obtained from 148 patients, and was correlated with clinicopathological parameters, molecular phenotypes, tumor cells’ proliferative capacity, and overall disease-free patients’ survival. Additionally, the expression of PXR was examined on human breast carcinoma cell lines of different histological grade, hormonal status, and metastatic potential. PXR positivity was noted in 79 (53.4%) and high PXR expression in 48 (32.4%), out of 148 breast carcinoma cases. High PXR expression was positively associated with nuclear grade (*p* = 0.0112) and histological grade of differentiation (*p* = 0.0305), as well as with tumor cells’ proliferative capacity (*p* = 0.0051), and negatively with luminal A subtype (*p* = 0.0295). Associations between high PXR expression, estrogen, and progesterone receptor negative status were also recorded (*p* = 0.0314 and *p* = 0.0208, respectively). High PXR expression was associated with shorter overall patients’ survival times (log-rank test, *p* = 0.0009). In multivariate analysis, high PXR expression was identified as an independent prognostic factor of overall patients’ survival (Cox-regression analysis, *p* = 0.0082). PXR expression alterations were also noted in breast cancer cell lines of different hormonal status. The present data supported evidence that PXR was related to a more aggressive invasive breast carcinoma phenotype, being a strong and independent poor prognosticator.

## 1. Introduction

Pregnane X Receptor (PXR), a member of the nuclear receptor (NR) superfamily, discovered in 1998 [1], represents a modular protein sharing common regions, a highly variable N-terminal domain, a conserved DNA binding domain (DBD), an H region (H), and a C-terminal ligand-binding domain (LBD) [2,3]. PXR is mainly expressed in liver and intestine, as well as in other tissues and organs [4,5,6,7,8]. PXR, upon ligand activation, forms a heterodimer with the Retinoid X Receptor (RXR) that binds to PXR response elements, located in the 5′-flanking regions of PXR target genes, resulting in their transcriptional activation. Three RXR genes exist, coding for RXR-α, -β, and -γ, all of which are able to heterodimerize with PXR. Each of the three genes can produce different RXR isoforms through the use of alternative promoters or splice sites [9].

PXR may also act as a gene silencer. Activation of PXR by ligands could result in the dissociation of co-repressors, such as the silencing mediator of retinoid and thyroid receptors (SMRT) and of the nuclear receptor co-repressor (NCoR), allowing the binding of the co-activators glucocorticoid receptor interacting protein (GRIP) and steroid receptor coactivator-1 (SRC-1) [10]. The biological implications of PXR activation include the homeostasis of numerous endobiotics, such as glucose, lipids, steroids, and bile acids [11,12,13,14], as well as regulating the response to the presence of several xenobiotics [15]. New roles for PXR have been identified in inflammatory bowel disease, vitamin D metabolism, and bone homeostasis [16,17,18]. PXR is also involved in pathways related with liver steatosis and fibrogenesis [19,20,21]. PXR activation results in regulation of drug-metabolizing enzymes and transporters transcription [2,22,23]. Thus, PXR is implicated in drug metabolism and drug-drug interactions, while knowledge concerning its genetic polymorphisms may help to understand the variations in human drug response and ensure safe drug use [24].

Breast cancer represents the most common malignancy and cause of cancer-related death amongst women [25]. Mammary tumors consist of an heterogeneous group of malignancies with varying molecular signatures, morphology, and clinical behavior [26]. Estrogen receptor (ER), progesterone receptor (PR), and human epidermal growth factor receptor 2 (HER2) define the prognosis, identify tumors for targeted therapy, and remain the sole established single-molecule biomarkers defining the minimum breast cancer pathology data set [27]. ER-targeted endocrine therapies are effective for the treatment of patients with ER-positive breast tumors, and tamoxifen is the most widely used endocrine anti-estrogen treatment [27].

PXR is involved in various cancer types, including breast, pancreatic, endometrial, ovarian, prostate, colon, liver, and esophageal cancer [28,29]. Dotzlaw et al. initially reported that PXR expression levels did not differ between breast malignant tumors and their adjacent matched normal tissues [4]. On the other hand, in other studies, either PXR mRNA or protein levels were detected in carcinoma tissues but not in non-neoplastic and stromal cells of breast tumors [30,31]. However, there are limited clinical data, so far, concerning the potential association of PXR expression with clinicopathological characteristics, molecular subtypes, tumor cells’ proliferative capacity [30]. Moreover, no data exist concerning PXR expression in relation with patients’ survival in invasive breast carcinoma. In view of the above considerations, the aim of the present study was to evaluate the immunohistochemical PXR expression in invasive breast carcinoma in association with multiple clinicopathological characteristics: ER, PR, and HER2 expression, tumor cells’ proliferative capacity, as well as overall disease-free patients’ survival. Additionally, the expression of PXR was examined on human breast carcinoma cell lines of different histological grade, hormonal status, and metastatic potential (MDA-MB231, MDA-MB468, MDA-MB453, MCF-7, T47D).

## 2. Patients and Methods

### 2.1. Patients

One hundred forty-eight invasive breast carcinoma specimens, obtained from an equal number of patients who underwent surgical resection due to breast cancer, were included in our study. The patients’ ages ranged from 33 to 85 years (mean 57 years). None of them received pre-operative radiation or chemotherapy. The institutional ethical committee of the Medical School of the University of Athens approved this study. Informed consent was signed by all patients in order to use, for research purposes, their biological samples and clinical data [32].

Haematoxylin and eosin staining was performed for routine histological examination. All cases were classified in accordance with World Health Organization criteria [33] and were classified as ductal or lobular. Nuclear grading was based on nuclear pleomorphism. Staging at the time of diagnosis was based on the TNM system [19]. The combined histological grade (1, 2, or 3) of infiltrating ductal and lobular breast carcinomas was obtained, according to the modified Scarff–Bloom–Richardson histological system and the guidelines suggested by Nottingham City Hospital pathologists [34]. The clinicopathological characteristics of the series are shown in Table 1.

The patients were followed up for a time interval of 2, up to 241, months with a mean survival time of 84.14 ± 49.11 months. Overall survival (OS) was defined as the time interval between the date of surgery and the date of death due to breast carcinoma or the last follow-up. Disease-free survival (DFS) was defined as the time interval between the date of surgery and the date of detection of recurrence or the date of last follow-up without recurrence for breast carcinoma. At the time of the last follow-up, 27 (18.2%) patients were dead of disease (DOD), 18 (12.2%) were alive with disease (AWD), and 103 (69.6%) were alive and disease-free (ADF). All patients received conventional postoperative treatment depending on the extent of the disease, including adjuvant chemotherapy, radiation therapy, and anti-estrogen therapy, when indicated, according to the consensus recommendations at the time [35].

### 2.2. Immunohistochemistry

Immunostainings for PXR were performed on formalin-fixed, paraffin-embedded tissue sections, using a commercially available mouse monoclonal anti-PXR (G-11, sc-48403) IgG1 primary antibody (Santa Cruz Biochemicals, Santa Cruz, CA, USA). Four μm thick tissue sections were deparaffinized, rehydrated, immersed in 3% H_2_O_2_ for 30 min, and microwaved at 750 W in 0.01 M citrate buffer (pH 6.0) for 15 min, and then, they left to cool down in TBS (Tris-buffered saline). Incubation with primary PXR antibody was performed for 1 h at room temperature (37 °C), at a dilution 1:100. The standard two-step peroxidase conjugated polymer technique (DAKO Envision kit, DAKO, Carpinteria, CA, USA) was then performed. At the next step, immunostainings were visualized with diaminobenzidine tetrahydrochloride solution (DAB; Sigma, Saint Louis, MO, USA). Sections were counterstained with Harris’ hematoxylin and mounted in Entellan (Merck, Darmstadt, Germany). Appropriate negative controls were performed by omitting the primary PXR antibody and/or substituting it with an irrelevant anti-serum. Pancreatic cancer tissue sections, with known enhanced PXR expression, were used as positive controls [36]. A mouse anti-human Ki-67 antigen; IgG1k antibody (clone MIB-1, Dakopatts, Glostrup, Denmark) was used to evaluate the tumor cells’ proliferative capacity, as previously described [36]. The expression of ER, PR, and HER2 was assessed immunohistochemically, as previously described [35].

### 2.3. Evaluation of Immunohistochemistry

Immunohistochemical evaluation was performed by counting at least 1000 tumor cells, in each case, by two independent observers (P.A., S.T.) blinded to the clinical data. Specimens were considered PXR-positive when more than 5% of tumor cells within the section were positively stained. PXR immunoreactivity was scored according to the percentage of positive tumor cells as 0: negative staining- 0–4% of tumor cells positive; 1: 5–24% of tumor cells positive; 2: 25–49% of tumor cells positive; 3: 50–100% of tumor cells positive, and its intensity as 0: negative staining, 1: mild staining; 2: intermediate staining; 3: intense staining. Finally, PXR expression was classified as low; if the total score was 0–2 and high; if the total score was ≥3. In this way, we ensure that each group has a more homogeneous and sufficient number of cases in order to be comparable with the other groups [36].

Staining for ER and PR was evaluated according to CAP/ASCO recommendations, i.e., ER and PR assays are considered positive if there are at least 1% positive tumor nuclei in the sample in the presence of the expected reactivity of internal and external controls [37]. The fraction of HER2 positive stained cells was scored according to CAP/ASCO guidelines [38]. Ki-67 immunoreactivity was classified, according to the percentage of positively stained breast cancer cells that exceeded the median percentage value, into two categories (below and over mean value), as previously reported [36].

### 2.4. Cell Lines

The MCF10A human mammary epithelial cell line was used as control (non-malignant breast epithelial cells). T47D and MCF7 are ER and PR positive, MDA-MB-453 is ER and PR negative, and Her2 positive. MDA-MB-468 is Triple Negative A (TNA). MDA-MB-231 is Triple Negative B (TNB). MCF7 cells were cultured in DMEM, l-glutamine (Gibco, Life Technologies CA, USA), T47D and MDA-MB-453 in RPMI 1640 medium GlutaMAX (Gibco, Life Technologies, CA, USA), supplemented with 10% FBS (fetal bovine serum) (Gibco, Life Technologies, CA, USA) and 1% penicillin-streptomycin. MDA-MB-468 and MDA-MB-231 were cultured in Leibovitz’s L-15 Medium (Gibco, Life Technologies, CA, USA) with 10% FBS. MCF10A cells were cultured in DMEM/F-12 (Gibco, Life Technologies, CA, USA) supplemented with 5% horse serum, 100 ng/mL cholera toxin, 20 ng/ml epidermal growth factor (EGF), 0.01 mg/ml insulin, 500 ng/ml hydrocortisone, and 1% penicillin-streptomycin. Cell cultures were incubated at 37 °C in a humidified atmosphere containing 5% CO2–95% air [39].

### 2.5. Western Blot

Protein extraction was performed using ice-cold RIPA buffer. Bradford assay (Bio-Rad) was used to assess protein concentration in the extracts. Proteins were resolved by electrophoresis in SDS-polyacrylamide gels (10%). Then, they were transferred to a nitrocellulose membrane (Macherey-Nagel, Düren Germany). The membrane was blocked for 1 h, at room temperature, in PBST with 5% nonfat milk and then with 5% bovine serum albumin incubated with primary antibodies overnight at 4 °C. Anti-PXR (1:500, sc-48403, Biotechnology Inc., Santa Cruz, CA, USA) and anti-b-Actin (1:1000 sc-8035; Biotechnology Inc., Santa Cruz, CA, USA) were used as primary antibodies. Horseradish peroxidase-conjugated secondary antibodies (Dako, CA, USA) were used at 1:5000 dilution. The detection of the immunoreactive bands was performed with the LumiSensor Chemiluminescent HRP Substrate kit (GenScript, NJ, USA). Relative protein amounts were evaluated by a densitometry analysis using ImageJ software. We used three independent samples for each group and β-actin for the normalization [40].

### 2.6. Statistical Analysis

The associations of PXR protein expression with clinicopathological variables, tumor cells’ proliferative capacity, and ER, PR, and HER2 protein expression were evaluated by chi-square test. The Kaplan-Meier method was applied to construct survival curves, and the log rank test was applied to compare the differences between the curves. To assess, at a multivariate level, the associations between the potential prognostic marker and overall disease-free patients’ survival, a Cox proportional-hazard regression model was developed. A p-value lower than 0.05 was considered as the limit of statistical significance. For all analyses SPSS for Windows Software was used (SPSS Inc., 2003, Chicago, IL, USA).

## 3. Results

**Figure 1:**
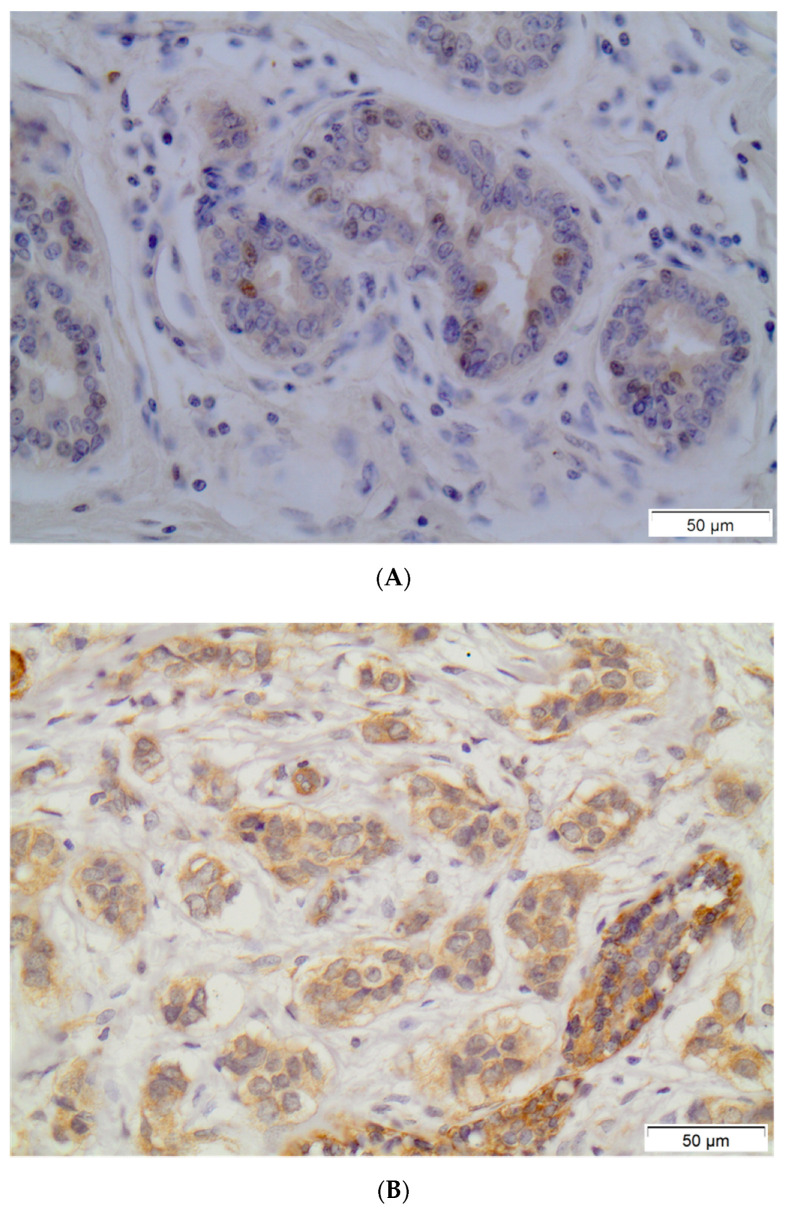
Representative PXR immunostainings in normal breast ductules (**A**) and in breast invasive carcinoma (**B**). Streptavidin-biotin-peroxidase, DAB chromogen, Harris hematoxylin counterstain (original magnification ×200, scale bars are present on figures).

PXR positivity (IHC score > 0) was noted in 79 (53.4%) out of 148 breast carcinoma cases. Out of the 148 examined samples, 48 (32.4%) presented high PXR expression (IHC score ≥ 3). The subcellular pattern of PXR distribution was mainly cytoplasmic and occasionally nuclear. Out of 79 PXR-positive breast cancer cases, 36 (45.6%) presented mild staining intensity, while 38 (48.1%) and 5 (6.3%) out of 79 PXR-positive breast carcinoma cases presented moderate or intense staining intensity, respectively. Normal surrounding areas adjacent to the tumor were found either negative or presented mild PXR nuclear immunostaining. Representative PXR immunostainings in breast carcinoma cells are depicted in Figure 1.

Out of 148 breast carcinoma cases, 65 (43.9%) were ER positive. PR positivity was noted in 60 (40.5%) out of 148 breast carcinoma cases, while 15 (10.1%) cases were HER2 positive. Out of 148 breast carcinoma cases, 59 (39.4%) were classified as luminal-A, 18 (12.1%) cases as luminal-B, 59 (9.4%) as triple negative, and 12 (8.0%) as HER2(+) phenotype.

In cross-tabulation, high PXR expression was significantly associated with high nuclear grade and high histological grade of differentiation (Table 1, *p* = 0.0112 and *p* = 0.0305, respectively). High PXR expression was significantly more frequently observed in ER(−) and PR(−) breast carcinoma cases (Table 1, *p* = 0.0314 and *p* = 0.0208, respectively). High PXR expression was significantly associated with increased tumor cells’ proliferative rate, assessed as Ki-67 protein statement (Table 1, *p* = 0.0051). Luminal-A subtype breast carcinoma cases presented a reduced incidence of high PXR expression compared to luminal-B, HER2, and triple negative molecular subtypes (Table 1, *p* = 0.0295).

**Figure 2:**
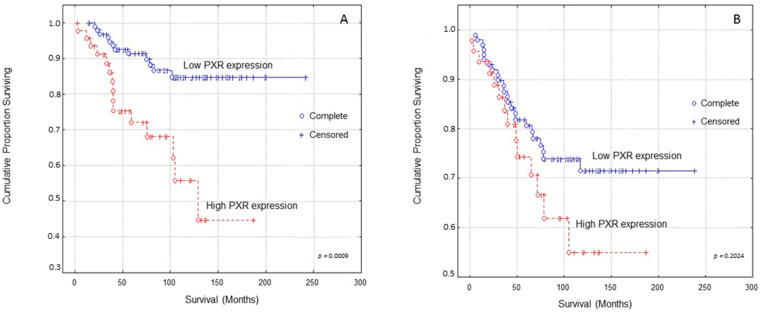
Kaplan–Meier survival analysis stratified according to PXR expression in 148 breast carcinoma patients for: (**A**) Overall patients’ survival and (**B**) Disease-free patients’ survival. Complete: When the patient completed the follow-up Censored: When the patient, for any reason, did not complete the follow-up.

Kaplan–Meier survival curves indicated that breast carcinoma patients presenting high PXR expression presented significantly shorter OS (Overall Survival) times compared to those with low PXR expression (Figure 2A, log-rank test, *p* = 0.0009). In multivariate analysis, PXR expression and Ki-67 protein statement were identified as independent prognostic factors of patients’ OS (Table 2, Cox-regression analysis, *p* = 0.0056 and *p* = 0.0007, respectively).

Kaplan–Meier survival curves indicated that breast carcinoma patients presenting high PXR expression showed shorter DFS (Disease-free survival) times compared to those with low PXR expression at a non-significant level (Figure 2B, log-rank test, *p* = 0.2024).

**Figure 3:**
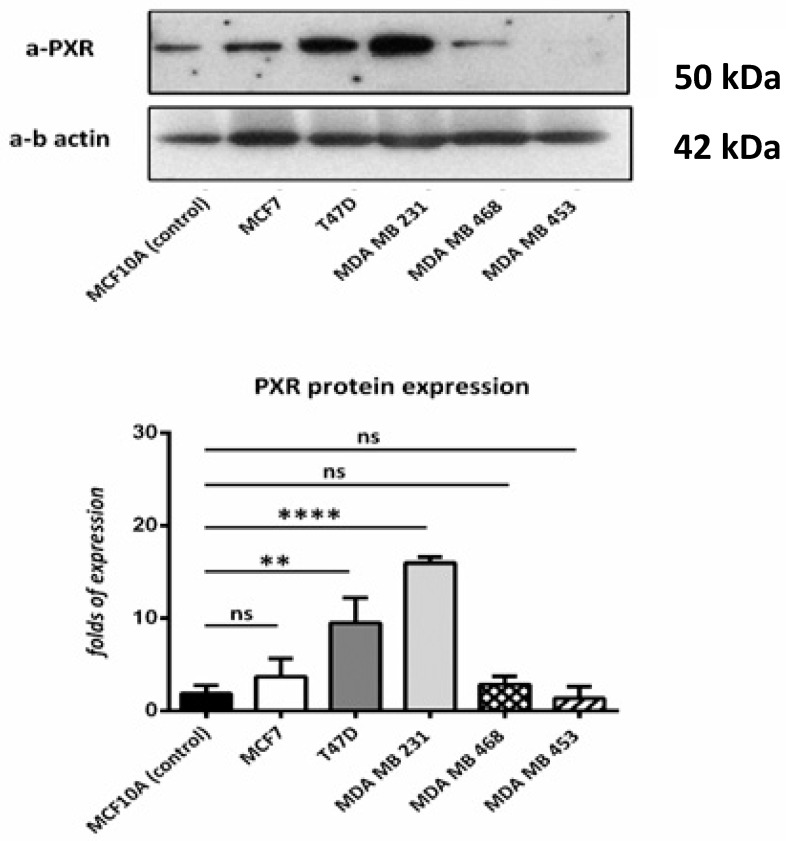
Western blot for PXR. We used MCF10A (as control) T47D, MCF7, MDA-MB-453, MDA-MB-468, and MDA-MB-231 cell lines. PXR is expressed in all the cell lines, despite the different molecular profile. Relative protein amounts were evaluated by a densitometry analysis using ImageJ software. We used three independent samples for each group and β-actin for the normalization. ns: no significant **: *p* < 0.01 **** *p* < 0.0001.

According to the immunoblotting results, all cell lines that were used show PXR expression in comparison with the control cell line. Higher expression was observed in T47D and MDA MB 231 cell lines. (Figure 3).

## 4. Discussion

PXR is involved in cancer in different ways, either by directly affecting cell proliferation and apoptosis or by inducing chemotherapy resistance, while PXR polymorphisms may also have clinical significance in certain cancer types and their treatment. Several mechanisms have been proposed for PXR-mediated effects in cancer and include regulation of genes involved in apoptosis, cell proliferation, angiogenesis, oxidative stress, cell cycle arrest, metabolism, inflammatory response, metastasis, drug metabolism and resistance, transport, and homeostasis (glucose, lipid, steroid) [29,41,42]. Currently, there are only 3 clinical studies that assessed PXR expression levels in breast malignant tumors, however, most of them did not examine their potential association with clinicopathological parameters and patients’ survival [4,30,31]. PXR expression has been reported in both ER(+) and ER(−) cancers, with some studies stating that its expression negatively correlates with ER status, while others stressed that higher PXR expression associated with higher survival rates in ER(+) breast cancer patients [43]. No significant correlations between PXR expression and PR status have been reported. Additionally, according to the immunoblotting results of our study, all the breast cancer cell lines (with different molecular profiles) express PXR.

The results of our study demonstrated that PXR expression, assessed immunohistochemically in histopathological samples of breast carcinoma patients, was correlated with crucial clinicopathological parameters for patients’ management and prognosis. Notably, high PXR expression was associated with increased nuclear and histological grade of differentiation, as well as with increased tumor cells’ proliferative rate. High PXR expression was also more frequently observed in ER and PR negative breast carcinoma cases. However, high PXR expression was detected in both ER(+) and ER(−) samples. This is consistent with previous studies, which stated that PXR promotes tumorigenesis through various mechanisms. In vitro studies [44] showed that PXR upregulates organic anion-transporting polypeptide 1 A2 (OATP1A2) in breast cancer cells, and thus, OATP1A2-mediated estrogen uptake, enhancing cancer growth. In ER(−) cancer tissues, on the other hand, estrogens can mediate disease progression by binding and activating PXR, as suggested by Pondugula and Mani [41]. Moreover, PXR was detected in carcinoma tissues but occasionally in non-neoplastic cells of the breast, which is in accordance to previous studies [30,31]. These findings suggested that high PXR expression was associated with a more aggressive breast carcinoma phenotype. In accordance to our study, a significant positive correlation between PXR expression and histological grade of differentiation, as lymph node status in breast carcinoma was also recorded. Furthermore, in ER(+) cases, PXR expression was positively correlated with tumor proliferative capacity, assessed by Ki-67 labelling index [30]. Several recent studies also investigated potential associations between PXR expression and various clinicopathological parameters in other cancer types [28]. More to the point, nuclear PXR was expressed in 80% of high grade dysplasia (HGD) versus 13% of low grade dysplasia (LGD) cases of patients with Barrett esophagus, supporting evidence that PXR expression might be able to separate HGD from LGD and non-dysplasia cases [45,46]. In addition, PXR was detected in 35 (24.8%) out of 141 epithelial ovarian carcinoma cases and was significantly associated with patients’ age, histological grade, ER-α and PR status [47]. Elevated PXR expression was significantly associated with advanced disease stage and increased tumor proliferative capacity, assessed by Ki-67 labelling index, in uterine carcinosarcoma, leiomyosarcoma, and endometrial stromal sarcoma [48]. In a previous study by our research group, pancreatic adenocarcinoma patients, presenting increased histological grade of tumor differentiation, showed a significant increased incidence of elevated PXR expression [36]. On the other hand, in human esophageal squamous cell carcinoma, nuclear PXR immunoreactivity was inversely correlated with histological grade, lymph node status, and Ki-67 labelling index [49].

Additionally, in our study, it was shown that breast carcinoma patients presenting high PXR expression showed significantly shorter OS and DFS times compared to those with low expression. Other studies have also investigated potential association between PXR expression and patients’ survival in different cancer types. In accordance with our study, PXR expression, assessed by real time quantitative polymerase chain reaction (RT-PCR), was inversely correlated with OS in invasive bladder carcinoma patients [50]. In epithelial ovarian carcinoma patients, a significant negative correlation between PXR-positive status and both DFS and OS was found [47]. Another study showed that nuclear distribution of PXR occurred in tissue samples from breast carcinoma patients who presented recurrence [31]. Moreover, no significant correlation between PXR expression, based on staining intensity and extent of positivity, and both DFS and OS in uterine sarcoma patients was noted [48]. On the contrary, cancer-specific survival in prostate cancer patients, presenting high PXR expression levels, based on staining intensity and extent of positivity, was significantly increased [51]. High nuclear PXR expression in the invasive front of the tumor area was significantly correlated with favorable clinical outcome of esophageal squamous cell carcinoma patients [49].

Notably, drug-mediated PXR activation can lead to undesired drug interactions and inducing chemotherapy resistance. PXR-mediated MRP2 induction seems to play a role in the additional acquisition of chemotherapy resistance in tamoxifen-resistant breast cancer [52,53]. Additionally, down-regulation of PXR expression caused a significant increase in endometrial cell growth inhibition and an enhancement of apoptosis in presence of the anticancer agents, paclitaxel and cisplatin [54,55,56]. PXR overexpression in colorectal cancer tissue samples led to a marked chemoresistance to the active metabolite of irinotecan [57,58,59]. Moreover, pre-treatment of osteosarcoma cells with the PXR antagonist ketoconazole, before exposure to etoposide, significantly increased the sensitivity of these cells to certain chemotherapeutic agents [60].

In view of the above findings, it should be speculated that the role of PXR in tumor growth and chemoresistance may vary depending on the specific cancer type and molecular signature of the cell line. Moreover, the above controversial results of PXR, observed in different cancer tissues, might be ascribed to the different PXR isoforms used. The HNF4a is a transcription factor related to PXR and is mainly located in the liver and intestine, participating in PXR induced signaling. In breast tissues HNF4a is not expressed. Nuclear receptors such as farnesoid X receptor (FXR), constitutive androstane receptor (CAR), peroxisome proliferator-activated receptor alpha (PPARα), liver X receptor (LXR), and androgen receptor, are candidates for the control of PXR expression. Additionally, possible coactivators that help the trafficking are steroid receptor coactivators (SRCs) 1, 2, and 3, peroxisome proliferator activated receptor gamma coactivator 1-alpha (PGC-1α), forkhead box O 1 transcription factor (FOXO1), protein arginine methyltransferase (PRMT), and p300 [61,62]. Furthermore, PXR may undergo epigenetic and post-translational modifications that affect its activity, so it may be selectively activated in some tumors [63].

As research results continue to reveal the complex role of PXR in neoplastic and metabolic diseases, it is increasingly capturing the interest as a therapeutic target candidate. Inhibition of PXR can have possible implications in the treatment of various malignancies and benign diseases, such as hepatic steatosis and diabetes.

## 5. Conclusions

The present study supported evidence that PXR expression in breast cancer was associated with crucial clinicopathological parameters for patients’ management and prognosis, supporting evidence for a potential important role of PXR in the biological mechanisms governing breast malignant disease progression. Moreover, this is the first study that examined the prognostic significance of PXR, documenting that PXR expression was an independent factor of poor prognosis in breast cancer. Better understanding the biology of breast cancer, the molecular pathways of cancer in which PXR is involved, and the correlation of PXR with drug resistance remain crucial steps in order to define the specific role of PXR in breast carcinogenesis.

## Figures and Tables

**Table 1:** Associations between PXR expression and clinicopathological parameters in 148 patients with invasive breast carcinoma. Statistically significant values are shown in bold.

Clinicopathological Parameters	PXR Expression		
	Low (%)	High (%)	*p*-Value
***N* = 148**	100 (67.6)	48 (32.4)	
Age (mean ± SD; ys)			0.8124
≤57.4 ± 12.5 yrs	50 (33.8)	25 (16.9)	
>57.4 ± 12.5 yrs	50 (33.8)	23 (15.5)	
Menopausal status			0.3032
Premenopausal	33 (22.3)	20 (13.5)	
Postmenopausal	67 (45.3)	28 (18.9)	
Histopathological type			0.6391
Ductal	67 (45.3)	34 (23.0)	
Lobular	33 (22.3)	14 (9.5)	
Histological Grade			**0.0305**
Low	13 (8.8)	3 (2.0)	
Intermediate	55 (37.2)	19 (12.8)	
High	32 (21.6)	26 (17.6)	
Nuclear Grade			**0.0112**
Low	51 (34.5)	12 (8.1)	
Intermediate	26 (17.6)	19 (12.8)	
High	23 (15.5)	17 (11.5)	
Molecular subtype			**0.0295**
Luminal-A	48 (32.4)	11 (7.4)	
Luminal-B	11 (7.4)	7 (4.7)	
HER2	6 (4.0)	6 (4.0)	
Triple negative	35 (23.6)	24 (16.2)	
Tumor size			0.6648
pT1	34 (23.0)	13 (8.8)	
pT2	55 (37.2)	30 (20.3)	
pT3	11 (7.4)	5 (3.4)	
Lymph nodes			0.9240
Non infiltrated	45 (30.4)	22 (14.9)	
Infiltrated	55 (37.2)	26 (17.5)	
Histopathological stage			0.9054
I	25 (16.9)	13 (8.8)	
II	60 (40.5)	29 (19.6)	
III	15 (10.1)	6 (4.0)	
ER expression			**0.0314**
Negative	50 (33.8)	33 (22.3)	
Positive	50 (33.8)	15 (10.1)	
PR expression			**0.0208**
Negative	53 (35.8)	35 (23.6)	
Positive	47 (31.8)	13 (8.8)	
HER-2 expression			0.6148
Negative	89 (60.2)	44 (29.7)	
Positive	11 (7.4)	4 (2.7)	
Ki-67 protein statement			**0.0051**
Below median value	62 (41.9)	18 (12.1)	
Over median value	38 (25.7)	30 (20.3)	

**Table 2:** Multivariate analysis for nuclear grade, histopathological stage, Ki-67 statement, and PXR expression for overall and disease-free patients’ survival. Statistically significant values are shown in bold.

Clinicopathological Variables	Overall Survival	
	HR (95% CI)	*p*-Value
Histological type (Ductal/Lobular)	0.677 (0.067–2.985)	0.4351
Nuclear Grade (Low/Intermediate + High)	0.582 (0.093–2.076)	0.2972
Histopathological stage (I + II/III)	1.908 (0.839–3.985)	0.1787
Ki-67 statement (Below/over median value)	6.173 (3.219–9.444)	**0.0007**
PXR expression (Low/High)	0.183 (0.022–0.589)	**0.0056**

## Data Availability

The data presented in this study are available on request from the corresponding author.
